# Credence Signals in Beef Consumption: The Strategic Role of the “100% Autochthonous Breed” Label in Spain

**DOI:** 10.3390/foods14142411

**Published:** 2025-07-08

**Authors:** Miguel A. Cantarero-Aparicio, José Manuel Perea, Alfonso Carbonero, Jennifer Claros-Zafra, Manuel Luque, Elena Angón

**Affiliations:** 1 Departamento de Producción Animal, Universidad de Córdoba, 14071 Cordoba, Spain; t42caapm@uco.es (M.A.C.-A.); pa2pemuj@uco.es (J.M.P.); v72clzaj@uco.es (J.C.-Z.); eangon@uco.es (E.A.); 2 Departamento de Sanidad Animal, Universidad de Córdoba, 14071 Cordoba, Spain; 3 Real Federación Española de Asociaciones de Ganado Selecto (RFEAGAS), C/Castelló 45, 2° Izda, 28001 Madrid, Spain; manuel.luque@rfeagas.es

**Keywords:** consumer preferences, autochthonous breed, beef, quality labels, conjoint analysis, cluster analysis, willingness to pay, food certification

## Abstract

This study evaluates the perceived value of the “100% Autochthonous Breed” label in beef purchasing decisions, comparing its influence with two well-established official certifications: organic and Protected Geographical Indication (PGI). A face-to-face survey was conducted with 900 consumers across Spain, using a choice-based conjoint experiment and hierarchical cluster analysis. The results indicate that although price is the primary determinant at the aggregate level, segmentation revealed distinct consumer profiles for whom the “100% Autochthonous Breed” label generated higher utility than other attributes. Specifically, four clusters were identified: Group I (18.6%) preferred the organic label; Group II (46.6%) prioritized low price; Group III (22.9%) valued the combination of PGI and the autochthonous breed label; and Group IV (11.9%) showed a preference for high-priced products featuring the “100% Autochthonous Breed” label. The findings highlight the strategic potential of this certification as a differentiation tool for sustainable, extensive, and territorially embedded livestock systems.

## 1. Introduction

Meat production and consumption are currently at the forefront of global debates concerning environmental sustainability, economic viability, and social equity [1,2]. Despite increasing criticism of the environmental footprint of the dominant food system, meat consumption remains high in developed countries and continues to rise in many developing economies [3]. Within this context, extensive livestock systems based on autochthonous breeds have re-emerged as a more sustainable alternative to prevailing intensive production models [4,5,6]. These systems are deeply rooted in rural territories and contribute to biodiversity conservation, ecosystem balance, and the maintenance of rural populations [7,8,9,10].

Local breeds are particularly well adapted to challenging environments and exhibit greater resistance to disease, thereby reducing dependency on external inputs due to more efficient use of local resources and increasing the resilience of production systems [7,11]. These attributes are strategically relevant in the context of climate change and the growing uncertainty regarding resource availability and input prices [5]. However, these production models face significant economic constraints, primarily due to higher cost structures and limited competitiveness in markets dominated by undifferentiated, low-cost meat products [12].

In response, quality signaling through product differentiation has become a key strategy for enhancing the competitiveness of sustainable livestock products [13,14]. Labelling enables the communication of credence attributes—such as animal welfare, production method, or geographical proximity—that are not directly observable by consumers, while also fostering trust, ensuring transparency, and addressing increasing ethical and environmental concerns [15,16].

In the European context, certification schemes such as Protected Geographical Indication (PGI), Protected Designation of Origin (PDO), and organic labels have become prominent tools for product valorisation and market segmentation [17,18]. However, in Spain, organic meat certification has not achieved the same level of market penetration as in other European countries. This limited uptake has been attributed to a combination of factors, including lower consumer trust, limited perceived sensory differences compared to conventional products, and price premiums that often exceed market acceptance thresholds [19,20].

In this context, the official “100% Autochthonous Breed” label—promoted by the Spanish Ministry of Agriculture, Fisheries and Food—represents a distinctive national initiative aimed at enhancing the value of Spain’s genetic livestock heritage [21]. This certification guarantees both the genetic purity and geographical origin of the breeds used in production and is typically associated with extensive grazing systems, environmental sustainability, territorial rootedness, and high animal welfare standards. With one of the most diverse catalogues of native breeds in Europe, Spain may find in this label a strategic opportunity to differentiate its meat products and strengthen consumer identification with regional values and traditional practices.

This study aims to analyze the perceived value of the “100% Autochthonous Breed” label in Spanish beef purchasing decisions. Using a choice-based conjoint analysis, the relative impact of the national label is assessed in comparison with two widely recognized European certifications: organic and PGI. Consumer profiles are subsequently segmented based on preferences and willingness to pay for each attribute, enabling the identification of consumer groups that are particularly responsive to the national label and providing insight into its positioning relative to established schemes.

This approach offers empirical evidence to inform more effective differentiation strategies for extensive livestock producers, thereby enhancing their competitiveness in a market increasingly shaped by demands for sustainability, traceability, and authenticity.

## 2. Materials and Methods

### 2.1. Study Design and Data Collection

This study was conducted in Spain between 2022 and 2023 with the objective of analyzing consumer preferences in beef purchasing decisions, with a specific focus on the perceived value of different quality certifications. To this end, a structured questionnaire was developed and administered in person by trained interviewers.

A non-probabilistic convenience sample was used, consisting of 900 consumers distributed across six Spanish geographic areas: Córdoba (*n* = 120), Jerez (*n* = 140), Madrid (*n* = 150), Santander (*n* = 110), Salamanca (*n* = 200), and Seville (*n* = 180). The sampling strategy aimed to approximate the demographic distribution of the national population in terms of key variables such as gender, age, and educational attainment [22]. Although this approach limits the statistical generalizability of the findings, it is deemed appropriate for exploratory research focused on identifying behavioural patterns and consumer profile differences [23,24,25]. To enhance the practical validity of the results, only participants with regular responsibility for household food purchases were included.

Surveys were conducted through face-to-face interviews in high-traffic locations, including shopping centres and supermarkets located in both urban and peri-urban areas. This broader territorial coverage allowed for a more realistic representation of the socioeconomic context in each study area and helps explain the relatively high percentage of respondents who reported living in rural areas. Interviews lasted an average of 15 min. Prior to full deployment, the questionnaire was pre-tested with 25 consumers to ensure clarity of language and comprehension of the experimental design, leading to minor adjustments.

The questionnaire was structured into two sections. The first collected sociodemographic data and information on meat consumption habits. The second included a choice-based conjoint experiment designed to estimate the relative importance of various beef certifications and consumers’ willingness to pay (WTP) for each.

Regarding demographic characteristics (Table 1), the sample showed a distribution broadly comparable to the Spanish population [22].

### 2.2. Conjoint Experiment Design

A discrete choice-based conjoint analysis was employed to deconstruct consumer preferences based on observable product attributes. Four key attributes were included: organic certification (present/absent), Protected Geographical Indication (PGI) (present/absent), the “100% Autochthonous Breed” label (present/absent), and price (with three levels: EUR 18, EUR 25, and EUR 32 per kilogram).

Price levels were derived from actual market prices observed shortly before data collection and represented a realistic range for beef loin cuts. The lowest price (EUR 18/kg) corresponded to uncertified products, the highest (EUR 32/kg) corresponded to products with at least one certification, and the intermediate value (EUR 25/kg) represented an average between both extremes. Rounded values were selected to facilitate comprehension and cross-profile comparisons.

Combining all attribute levels produced a total of 24 possible product profiles. To reduce cognitive load and eliminate multicollinearity among attributes, an orthogonal fractional factorial design was applied, resulting in a reduced and statistically efficient set of 8 profiles (Table 2). Such designs are commonly used in discrete choice studies to estimate main effects with fewer combinations [26,27].

Each participant completed 10 forced-choice tasks, selecting their preferred option among three alternative beef labels. This format, which excluded a “none of the above” option, allowed for more accurate estimation of relative preferences by simulating a realistic purchasing environment.

Before beginning the experiment, respondents were provided with a brief written explanation of each attribute and its levels, accompanied by visual examples. Responses were analyzed using conditional utility models.

### 2.3. Statistical Analysis

Data were analyzed using the conjoint analysis module in XLSTAT [28]. This tool estimates part-worth utilities for each attribute level via ordinary least squares (OLS) regression, with coefficients interpreted as the relative value assigned by consumers to each product characteristic [27]. From these results, individual-level utility scores were generated to reflect personal preferences across the tested attributes.

Willingness to pay (WTP) for each attribute was calculated by dividing the utility estimate of the attribute by the price coefficient. This method yields a monetary estimate of the premium consumers are willing to pay for the presence of a given certification [29].

To identify homogenous consumer segments based on their preference patterns, a hierarchical cluster analysis was performed using individual-level utilities. Squared Euclidean distance was used as the proximity metric, and Ward’s method was employed for clustering, which minimizes within-group variance [30,31].

The optimal number of clusters was determined using the elbow criterion, examining the plot of explained inertia relative to the number of clusters. The point at which the marginal gain from adding additional clusters diminished was selected. This decision was further validated using the Calinski–Harabasz index, which compares between- and within-cluster variance and confirmed the appropriateness of the selected clustering solution [26,32,33].

Once clusters were defined, differences in preference patterns across groups were examined. Variations in relative utilities, attribute importance, and WTP were analyzed using one-way analysis of variance (ANOVA). When ANOVA results were significant, the Student–Newman–Keuls (SNK) post hoc test was applied to identify pairwise differences between clusters. Additionally, the sociodemographic characteristics of each cluster were examined using chi-square (χ^2^) independence tests to explore whether segmentation by preferences was also associated with structural demographic differences. All statistical analyses were conducted using XLSTAT software (Version 2024.2) [28].

## 3. Results

### 3.1. Aggregate Results of the Conjoint Analysis

The conjoint analysis results reveal that Spanish beef consumers exhibit a clear preference for products carrying differentiated quality attributes, such as those included in this study (Table 3). In terms of part-worth utilities, the three certifications analyzed—“100% Autochthonous Breed”, PGI, and organic production—produced positive utilities when present and negative utilities when absent, indicating that they contribute positively to perceived consumer value.

Price emerged as the most influential attribute in consumer choice, with a relative importance of 33.8%. As expected, lower price levels were strongly preferred, while higher-priced options were penalized. This price sensitivity suggests that, although consumers appreciate differentiating attributes, cost remains the primary determinant in beef purchasing decisions.

On a secondary level, and with very similar weightings, the three certifications showed comparable importance: organic certification (22.6%), “100% Autochthonous Breed” label (22.6%), and PGI (21.0%). In all cases, the presence of the respective label resulted in positive utility values and was preferred over its absence.

In terms of WTP, consumers expressed a moderate but non-negligible willingness to pay a premium of approximately EUR 1/kg for each of these three quality attributes. The organic and PGI labels generated slightly higher utility values than the autochthonous breed label, although the differences were not statistically significant at the aggregate level.

### 3.2. Consumer Segmentation Through Cluster Analysis

Based on the individual utility patterns obtained from the conjoint analysis, a hierarchical cluster analysis was performed to identify distinct consumer segments according to their preference structures. This procedure yielded four homogeneous groups, each exhibiting statistically significant differences in the relative importance attributed to the various attributes (Table 3). One-way ANOVA revealed statistically significant differences (*p* < 0.05) across clusters for all attributes analyzed. In contrast, the analysis of sociodemographic characteristics across segments (Table 4) showed no significant differences, suggesting that the segmentation reflects attitudinal rather than demographic variables.

Cluster I—Green-oriented consumers

This segment prioritized the organic certification attribute, assigning it a relative importance of 39.4% (the highest among all attributes for this group). Price (26.3%) and PGI (22.5%) followed in relevance, while the “100% Autochthonous Breed” label was the least valued attribute (11.7%). Consumers in this group demonstrated a particularly high WTP for organic certification (EUR 2.56/kg), which was the highest across all clusters, and a near-negligible WTP for the autochthonous breed label (EUR 0.47/kg). This profile aligns with environmentally and health-conscious consumers who are moderately price-sensitive and prioritize production methods over origin or breed.

Cluster II—Price-sensitive consumers

This group showed a clear preference for low-priced products (EUR 18/kg), with a relative importance of 42.8%, the highest among all groups. The remaining attributes (autochthonous breed, organic, and PGI) were valued similarly, with relative importance values between 18% and 20%. WTP for quality certifications was low or nearly null: EUR 0.43/kg for both organic and autochthonous breed labels, suggesting a pragmatic attitude in which price is the principal decision criterion. This profile represents functional consumers who value quality only when it does not entail a significant price premium.

Cluster III—Local-oriented consumers

Consumers in this group strongly valued the “100% Autochthonous Breed” label (34.1%) and the PGI certification (24.9%), reflecting a preference structure centered on geographical origin and ties to traditional livestock practices. Their WTP was particularly high for the autochthonous breed (EUR 5.89/kg) and PGI (EUR 3.52/kg), making them the cluster with the highest WTP for these attributes. Conversely, they showed lower price sensitivity. This segment likely represents consumers committed to local production, authenticity, and territorial support.

Cluster IV—Premium consumers

Members of this group favored high-priced products (EUR 32/kg) and the autochthonous breed attribute (32.7%), both of which had high relative importance values. In contrast, organic and PGI certifications were rated as low priority, making this the cluster that valued these two attributes the least. Their WTP for the “100% Autochthonous Breed” label was relatively high (EUR 1.62/kg), while their WTP for other attributes was marginal. This profile corresponds to high-end consumers who are less price-sensitive and associate autochthonous breeds with prestige, tradition, and premium differentiation.

## 4. Discussion

This study examined the preferences of Spanish beef consumers in relation to various extrinsic attributes: price, organic certification, PGI, and the “100% Autochthonous Breed” label. Through conjoint analysis and subsequent cluster segmentation, the results highlight, on the one hand, the predominant influence of price in consumer choices and, on the other, the existence of clearly differentiated consumer segments based on the relative importance assigned to each attribute and their corresponding WTP. While the three credence attributes showed similar aggregate value, the cluster analysis revealed that preference hierarchies vary significantly across segments, with the autochthonous breed label emerging as the most valued attribute for some groups. Although the overall WTP was modest, certain segments (particularly those sensitive to territorial identity) exhibited notably high premiums for this label.

Consistent with previous studies on meat and other food products [34,35,36], price was the most influential attribute, with a relative importance of 33.8%, well above that of the other factors. This price sensitivity was particularly evident in the “price-sensitive” cluster, wherein certifications had minimal impact. However, the presence of consumer segments that prioritize non-price attributes underscores the feasibility and necessity of differentiation strategies in a heterogeneous market.

At the aggregate level, the three certifications (organic, PGI, and “100% Autochthonous Breed”) displayed similar levels of importance (between 21% and 23%). This apparent equilibrium suggests that there is no single dominant attribute in shaping consumers’ perception of quality. Rather, each certification signals a distinct but complementary dimension: organic certification conveys environmental sustainability and animal welfare [13,37,38,39]; PGI guarantees geographic origin and production tradition [40,41]; and the autochthonous breed label affirms genetic purity and a connection to local livestock biodiversity [21,42]. Although these are distinct credence attributes, they often converge in the consumer’s perception, forming a signaling system that reinforces product authenticity and differentiation [20,43,44,45,46].

Nonetheless, the cluster analysis shows that this global symmetry conceals distinct hierarchies within each segment, where one or another attribute may assume a dominant role. This finding underscores the importance of market segmentation and the need to tailor communication strategies to the priorities and sensitivities of specific consumer profiles [47].

Cluster analysis identified four clearly differentiated consumer segments. The green-oriented segment prioritized production methods and preferred organic meat, though without a correspondingly high WTP [40], reinforcing previous observations in Spain that the symbolic value of the organic label does not always translate into economic behavior [18,20].

In contrast, the “localist” group exhibited a strong WTP for the autochthonous breed label, along with high valuations for PGI and organic attributes. This pattern suggests heightened sensitivity to products with territorial and traditional ties [14]. The “premium” segment combined a preference for high-priced products with a clear appreciation for the autochthonous breed label, suggesting that this certification may function as a marker of prestige and exclusivity.

Conversely, the price-sensitive group largely disregarded quality certifications, highlighting the importance of adapting commercial strategies to different levels of economic sensitivity. Across all segments, no statistically significant differences were found in sociodemographic variables, aligning with previous studies that indicate that attitudes, values, and beliefs are more explanatory than conventional demographic profiles [48,49].

One of this study’s main contributions is the empirical analysis of the “100% Autochthonous Breed” label, an official certification by the Spanish Ministry of Agriculture [21], which had not previously been assessed from the consumer perspective. The results demonstrate that this label can generate both utility and willingness to pay, especially among segments that value origin, authenticity, and traditional livestock systems.

In the “localist” cluster, the label was the most important attribute, surpassing even price, while in the “premium” group, it functioned as a symbol of distinction. These findings contrast with the marginal treatment that breed-related factors typically receive in food labelling [25,50] and support the view that breed can act as a credible marker of quality, tradition, and sustainability when communicated effectively.

However, some methodological limitations must be acknowledged. In the experimental design, respondents were given a clear and concise explanation of each label’s meaning, including the autochthonous breed logo. This artificially increases the level of information compared to real-world shopping environments, where consumers often lack knowledge or confuse certification schemes [20]. Under real market conditions, the impact of this label might be lower unless supported by effective communication campaigns.

Moreover, although the label certifies the genetic purity of specific breeds, the experiment did not specify any particular breed, allowing respondents to project their own associations (e.g., familiar, local, or idealized breeds) [51]. While this may have increased the label’s perceived value through “positive ambiguity”, it also introduces uncontrolled interpretive variability, suggesting that the results might differ if the same label were evaluated in association with specific breeds.

Another relevant insight from this study concerns the conceptual overlap among the extrinsic attributes assessed. PGI, organic, and autochthonous breed certifications share overlapping associations with origin, sustainability, and product differentiation [6,14,52,53]. In some segments, particularly the “localist” group, these attributes appear to function as complementary or even partially substitutable cues [54], complicating the consumer’s ability to discern the unique value each one adds.

This overlap has strategic implications. On the one hand, combining multiple labels on the same product may generate synergistic effects that enhance overall quality perception [14,53,55,56,57]. On the other, it may dilute the individual effectiveness of each certification if consumers struggle to clearly differentiate their meaning [58,59,60,61]. These findings underscore the need for differentiated, clear, and educational communication by all actors involved in quality certification.

## 5. Conclusions

This study advances understanding of consumer behavior in relation to extrinsic attributes in beef purchasing decisions by providing the first empirical analysis of the official “100% Autochthonous Breed” label. The findings highlight the strategic potential of this certification as a differentiation tool in a market currently dominated by more established schemes such as PGI and organic labels. Although its market penetration remains limited, the label demonstrates an ability to generate perceived value and willingness to pay among specific consumer segments, positioning it as a key asset for enhancing the value of sustainable, extensive, and territorially rooted livestock systems.

At the aggregate level, price was the dominant determinant of consumer choice, whereas the three evaluated quality labels (organic, PGI, and “100% Autochthonous Breed”) were perceived as less influential and relatively similar in importance, without any one standing out markedly above the others. However, segmentation of the sample through cluster analysis revealed four distinct consumer profiles, each with specific preference patterns. Group I (18.6%) displayed a clear inclination toward organic certification. Group II, the largest segment (46.6%), prioritized low price as their main purchasing criterion. Group III (22.9%) placed higher value on the combined presence of the “100% Autochthonous Breed” label and the PGI certification, suggesting an appreciation for origin-based and culturally rooted quality indicators. Finally, Group IV (11.9%) showed a preference for premium-priced products, also demonstrating interest in the “100% Autochthonous Breed” label, highlighting a niche segment motivated by perceived exclusivity and product differentiation. The main practical implication of these results lies in the need to develop more segmented and pedagogical communication strategies capable of clearly conveying the specific value associated with each certification. Perceived overlaps between labels (such as PGI, organic, and autochthonous breed) may reduce their effectiveness if their distinct meanings are not clearly articulated. Moreover, the results reinforce the notion that the consumer base is not homogeneous, but rather composed of diverse profiles with varying motivations, priorities, and barriers. This heterogeneity necessitates tailored marketing approaches in terms of messaging, language, and distribution channels for each target segment.

Future research could explore how consumer perceptions of the “100% Autochthonous Breed” label vary when it is explicitly linked to specific breeds, as well as its interaction with other co-occurring certifications on the same product. Additionally, evaluating its impact on actual purchase behavior in real commercial settings (both physical and digital) would offer valuable insights. Lastly, further investigation is warranted into the role of territorial narratives, emotional attachment to origin, and institutional trust in shaping the perceived value of public certifications.

## Figures and Tables

**Table 1:** Demographic characteristics of consumers and the Spanish population (*n* = 900).

Variable	Global (%)	Spanish National Population ^a^ (%)
Gender		
Female	34.7	51.0
Male	65.3	49.0
Area of Residence		
Urban zone	56.4	81.2
Rural zone	43.6	18.8
Age group (in years)		
18–24	9.3	7.8
25–34	9.3	12.5
35–49	20.3	24.7
50–64	37.3	22.9
≥65	23.7	32.1
Family size		
1	6.8	26.3
2	26.3	30.4
3	22.9	17.3
4	33.0	17.1
5 or more	11.0	8.9
Employment status		
Student	9.4	6.1
Public employee	19.7	16.5
Retired	15.4	22.4
Private employee	35.0	74.6
Self-employed	20.5	16.1
Monthly family net income		
<1000 EUR	3.7	14.3
1001–2000 EUR	14.7	24.5
2001–3000 EUR	17.4	19.8
3001–4000 EUR	24.8	14.2
>4000 EUR	39.4	27.2
Education		
No studies	0.9	7.8
Basic education	6.0	26.5
Professional	14.7	24.3
University	77.6	29.1
Superior	0.9	12.3
Meat consumption		
1 time per week	14.3	8.9
2/3 times per week	51.8	14.7
4/5 times per week	20.4	21.3
Every day	3.6	55.1
3001–4000 EUR	24.8	14.2
>4000 EUR	39.4	27.2

^a^ National Statistics Institute, 2024 [22].

**Table 2:** Card profile obtained from the orthogonal design.

Option	Price (EUR/kg)	Geographical Indication (PGI)	Production Method (Organic Label)	Breed Label (100% Autochthonous Breed)
1	25	Yes	No	Yes
2	32	Yes	Yes	No
3	18	Yes	Yes	Yes
4	25	No	Yes	No
5	18	No	No	No
6	32	No	No	Yes
7	18	No	Yes	Yes
8	18	Yes	No	No

**Table 3:** Relative importance (%), utility values, and changes in willingness to pay (EUR/kg) for each attribute for groups of consumers obtained after clustering.

Variable	Global	Group			
		I	II	III	IV
100% Autochthonous-breed label					
Yes	0.90 ± 0.75	0.37 ^a^	0.86 ^b^	1.59 ^c^	0.60 ^ab^
No	−0.90 ± 0.75	−0.37 ^a^	−0.86 ^b^	−1.59 ^c^	−0.60 ^c^
Relative importance (%)	22.55 ± 13.79	11.73 ^a^	18.62 ^b^	34.11 ^c^	32.69 ^c^
WTP (EUR/kg)	−0.91 ± 0.14	−0.47	−0.43	5.89	1.62
PGI label					
Yes	1.17 ± 0.55	1.17 ^b^	0.98 ^b^	1.19 ^b^	−0.20 ^a^
No	−1.17 ± 0.55	−1.17 ^b^	−0.98 ^b^	−1.19 ^b^	0.20 ^a^
Relative importance (%)	21.01 ± 10.80	22.51 ^ab^	19.60 ^ab^	24.90 ^b^	16.71 ^a^
WTP (EUR/kg)	−1.18 ± 1.05	−2.56	−0.43	3.52	0.16
Organic label (%)					
Yes	1.00 ± 0.88	2.02 ^c^	0.86 ^b^	0.95 ^b^	0.06 ^a^
No	−1.00 ± 0.88	−2.02 ^c^	−0.86 ^b^	−0.95 ^b^	−0.06 ^a^
Relative importance (%)	22.60 ± 14.81	39.41 ^b^	19.03 ^a^	20.92 ^a^	13.46 ^a^
WTP (EUR/kg)	−1.01 ± 0.03	−2.56	−0.43	3.52	0.16
Price (EUR/kg)					
18	0.83 ± 1.18	−0.05 ^a^	1.65 ^b^	0.36 ^a^	−0.07 ^a^
25	0.16 ± 0.74	0.84 ^d^	0.38 ^c^	−0.63 ^c^	−0.29 ^b^
32	−0.99 ± 1.27	−0.79 ^b^	−2.02 ^a^	0.27 ^c^	0.37 ^c^
Relative importance (%)	33.83 ± 14.83	26.35 ^a^	42.75 ^c^	20.06 ^a^	37.13 ^b^
Size of cluster (%)		18.6	46.6	22.9	11.9

^a^, ^b^, ^c^, ^d^: means with different letters are significantly different between clusters (SNK test, *p* < 0.05).

**Table 4:** Sociodemographic characteristics for groups of consumers.

Variable	Global	I	II	III	IV	*p*-Value
Gender						ns
Female	34.7	40.9	38.2	25.9	21.4	
Male	65.3	59.1	61.8	74.1	78.6	
Area of Residence						ns
Urban zone	56.4	59.1	60.0	51.8	50.0	
Rural zone	43.6	40.9	40.0	48.1	50.0	
Age group (in years)						ns
18–24	9.3	9.1	10.9	11.1	0.0	
25–34	9.3	4.5	12.7	7.4	7.1	
35–49	20.3	18.2	20.0	29.6	7.1	
40–64	37.3	36.4	40.0	29.6	37.3	
65	23.7	31.8	16.4	22.2	42.9	
Family size						ns
1	6.8	4.5	10.9	3.7	0.0	
2	26.3	31.8	18.2	22.2	57.1	
3	22.9	22.7	27.3	22.2	7.1	
4	33.0	27.3	30.9	44.4	28.6	
5 or more	11.0	13.6	12.7	7.4	7.1	
Employment status						ns
Student	9.4	14.3	9.1	11.1	0.0	
Civil servant	19.7	19.1	18.2	33.3	0.0	
Retired	15.4	23.8	10.9	14.8	21.4	
Businessman	35.0	38.1	34.5	22.2	57.1	
Self-employed	20.5	4.8	27.3	18.5	21.4	
Monthly family net income						ns
<1000 EUR	3.7	5.0	1.9	8.3	0.0	
1001–2000 EUR	14.7	10.0	19.2	8.3	15.4	
2001–3000 EUR	17.4	20.0	13.5	25.0	15.4	
3001–4000 EUR	24.8	25.0	25.0	33.3	7.7	
>4000 EUR	39.4	40.0	40.4	25.0	61.5	
Education						ns
No studies	0.9	0.0	1.8	0.0	0.0	
Basic education	6.0	4.8	7.4	3.7	7.1	
Professional	14.7	14.3	16.7	14.8	7.1	
University	77.6	80.9	74.1	77.8	85.7	
Superior	0.9	0.0	0.0	3.7	0.0	
Meat consumption						ns
1 time per week	14.3	4.8	11.8	14.8	38.5	
2/3 times per week	51.8	61.9	49.0	55.6	38.5	
4/5 times per week	30.4	23.8	37.2	25.9	23.1	
Every day	3.6	9.5	2.0	3.7	0.0	

ns = non-significant (*p* > 0.05).

## Data Availability

The data presented in this study are available from the corresponding author upon request. The data are not publicly available due to project IP rules.
